# Impact of laparoscopic gastrectomy on long‐term prognosis of patients with primary T3 or more advanced gastric cancer: A propensity score matching analysis

**DOI:** 10.1002/ags3.12651

**Published:** 2022-12-29

**Authors:** Ryota Matsui, Noriyuki Inaki, Toshikatsu Tsuji

**Affiliations:** ^1^ Department of Gastroenterological Surgery Ishikawa Prefectural Central Hospital Kanazawa Japan; ^2^ Department of Gastrointestinal Surgery/Breast Surgery, Graduate School of Medical Science Kanazawa University Kanazawa Japan

**Keywords:** gastric cancer, laparoscopic gastrectomy, overall survival, prognosis, relapse‐free survival

## Abstract

**Background:**

There is no consensus regarding a better long‐term prognosis with laparoscopic gastrectomy than with open surgery in patients with advanced gastric cancer, especially in patients with T3 or more advanced gastric cancer. We investigated the impact of laparoscopic gastrectomy on the long‐term prognosis of patients who underwent radical gastrectomy for primary T3 or more advanced gastric cancer.

**Methods:**

This single‐center, retrospective cohort study included 294 consecutive patients who underwent radical gastrectomy for primary T3 or more advanced gastric cancer from April 2008 through April 2017. We compared overall survival between laparoscopic and open surgery, using propensity score matching to adjust for baseline characteristics. We also investigated prognostic factors for overall survival by a forward stepwise procedure of Cox proportional hazards regression for multivariate analysis.

**Results:**

There were 136 (46.3%) and 158 (53.7%) patients in the laparoscopy and open groups, respectively. The median follow‐up period was 39 mo. After matching, there were 97 patients in each group, with no significant differences in background characteristics. After matching, the overall survival was significantly worse in the open group than in the laparoscopy group (*P <* 0.001). Multivariate analyses also showed that open surgery was an independent poor prognostic factor for overall survival (hazard ratio: 2.160, 95% concordance interval: 1.365–3.419, *P =* 0.001).

**Conclusion:**

Overall survival may be better with laparoscopic gastrectomy than with open surgery for patients with primary T3 or more advanced gastric cancer.

## Graphical abstract

We investigated the impact of laparoscopic gastrectomy on the long‐term prognosis of patients who underwent radical gastrectomy for primary T3 or more advanced gastric cancer. After propensity score matching, the overall survival was significantly worse in the open group than in the laparoscopy group (P < 0.001). Multivariate analysis also showed that open surgery was an independent poor prognostic factor for overall survival (hazard ratio: 2.160, 95% concordance interval: 1.365‐3.419, P = 0.001).

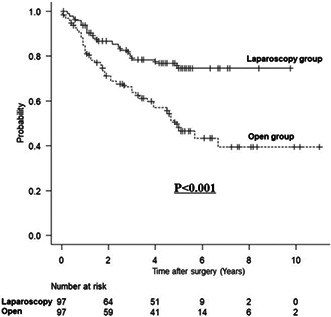

## 1 INTRODUCTION

There is no consensus regarding a better long‐term prognosis with laparoscopic gastrectomy than with open surgery in patients with advanced gastric cancer, especially in patients with T3 or more advanced gastric cancer. Among randomized controlled trials (RCTs) conducted in Asia, the CLASS‐01 trial comparing laparoscopic and open distal gastrectomies and demonstrated noninferiority in terms of 3‐y disease‐free survival,^1^ while the KLASS‐02 trial demonstrated noninferiority in terms of 3‐y relapse‐free survival when patients were stratified by pathological stage.^2^ It was recently reported that the 5‐y overall survival (OS) did not differ between the laparoscopic and open groups in the CLASS‐01 trial.^3^ Other studies using propensity score matching (PSM) showed no difference in the long‐term prognosis between the laparoscopic and open groups.^4^, ^5^, ^6^ In addition, the results of the JLSSG0901 study, an ongoing prospective, randomized, phase II study comparing laparoscopy‐assisted distal gastrectomy with D2 lymph node dissection and open surgery for locally advanced gastric cancer in Japan, are awaited.^7^

The purpose of this study was to investigate the impact of laparoscopic gastrectomy on the long‐term prognosis of patients who underwent radical gastrectomy for primary T3 or more advanced gastric cancer. The hypothesis was that laparoscopic surgery results in similar postoperative outcomes as with open surgery in patients with advanced gastric cancer.

## 2 MATERIALS AND METHODS

### 2.1 Study design

This single‐institution, retrospective cohort study was conducted at Ishikawa Prefectural Central Hospital, and it included consecutive patients who underwent radical gastrectomy with D2 lymph node dissection for primary T3 or more advanced gastric cancer, diagnosed according to the 15th edition of the Japanese Classification of Gastric Carcinoma, from April 2008 through April 2017. Some eligible patients in this study were enrolled in the JLSSG0901 study, of which 15 were in the open group and 16 in the laparoscopic group.^7^ We retrospectively collected clinical and laboratory data, including medical records and images, using the hospital's electronic patient record system. The exclusion criteria were as follows: residual gastric cancer, cancer involving other organs, preoperative treatment, adjuvant chemotherapy with regimens other than S‐1, and p‐stage IV. To avoid the influence of chemotherapy transition, we restricted our analysis to patients receiving S‐1 adjuvant chemotherapy. The background characteristics of patients were adjusted using PSM, following which the postoperative outcomes were compared. The study protocol was approved by the Institutional Ethical Review Committee of Ishikawa Prefectural Central Hospital (authorization number: 1589). The opt‐out recruitment method was applied to provide all patients an opportunity to refuse participation.

### 2.2 Indications for the procedure

Each patient underwent radical gastrectomy with D2 nodal dissection. Laparoscopic surgery or open surgery were indicated for all advanced gastric cancers. The surgeon selected the surgical approach, which was not changed throughout the procedure. For quality control, only one surgeon, who was a professional in gastrectomy and responsible for the quality of the laparoscopic surgery, was involved in this study, as in the JCOG‐0912 study protocol. The procedure for nodal dissection and reconstruction was the same in both groups.

### 2.3 Postoperative management with S‐1 adjuvant chemotherapy

Postoperative adjuvant chemotherapy with S‐1 was administered to patients with stage II and stage III disease for a maximum of 1 y, according to the Japanese gastric cancer treatment guidelines.^8^ The regimen was initiated with 80–120 mg/d for 4 weeks, followed by 2 weeks of rest. If side effects were observed, the dose was gradually reduced from 120 mg/d to 100 mg/d or from 100 mg/d to 80 mg/d, according to the guidelines. The treatment was discontinued if the side effects could not be controlled with dose optimization or two or more steps of dose reduction, or if recurrence of disease was confirmed during adjuvant chemotherapy. For recurrent cases, additional chemotherapy was administered according to the Japanese gastric cancer treatment guidelines.^8^ Patients did not receive any treatment other than adjuvant chemotherapy with S‐1 until recurrence.

The patients were followed up at an outpatient clinic. Hematological tests were performed at least every 2–3 weeks during S‐1 chemotherapy and at least every 3 mo for 5 y after chemotherapy completion. Computed tomography (CT) was performed every 6 mo, while endoscopy was performed every year until 5 y after surgery.

### 2.4 Body composition analysis

Before surgery, we measured the visceral fat area and skeletal muscle mass on plain CT images using the graphical analysis software Ziostation (Ziosoft, Newark, CA, USA). Visceral fat mass was measured at the umbilical level, and skeletal muscle mass was measured at the third lumbar vertebral level. Visceral fat mass and skeletal muscle mass measured on a single CT image slice were divided by the patient's height in meters squared to obtain the visceral adipose tissue index (VAI) and skeletal muscle mass index (SMI), respectively.^9^ The cutoff values for VAI and SMI were set separately for men and women using the cutoff values previously reported for Asians; the cutoff values for VAI were 35.43 cm^2^/m^2^ for men and 24.85 cm^2^/m^2^ for women, while those for SMI were 40.80 cm^2^/m^2^ for men and 34.90 cm^2^/m^2^ for women.^9^, ^10^, ^11^ Patients with VAI and SMI values above and below the cutoff values were considered to have high and low VAI/SMI, respectively.

### 2.5 Outcomes

The primary outcome was OS, defined as the period between surgery and death. The secondary outcomes were relapse‐free survival (RFS), cancer‐specific survival (CSS), and other‐cause survival (OCS). RFS was defined as the period between surgery and recurrence or death, whichever occurred first. Preoperative chronic inflammation was defined by a C‐reactive protein (CRP) level of ≥0.5 mg/dL just before the surgery, and OS was compared between laparoscopic and open surgery in patients with or without preoperative chronic inflammation.

Postoperative complications were defined as complications of grade ≥2 according to the Clavien–Dindo classification (CD) that occurred within 30 d after surgery. Complications of CD grade ≥3 were considered severe complications. The body weight loss (BWL) rate was calculated for 1 mo, 6 mo, and 1 y.

### 2.6 Statistical analyses

The PSM method was used to adjust for differences in the background characteristics of patients and selection bias in this nonrandomized study. A logistic regression model including age, sex, clinical stage, surgical procedure, comorbidities, and preoperative CRP as factors was used to estimate the propensity score, and 1:1 matching between the two groups was achieved using the nearest‐neighbor matching method. The caliper size was 0.20. Postoperative outcomes were compared between the two groups after matching.

For continuous variables, we used the Mann–Whitney *U*‐test, and for categorical variables, we used the Chi‐square test or Fisher's exact test. The log‐rank test was used for long‐term survival. We used a forward stepwise procedure of Cox proportional hazards regression for multivariate analysis to identify prognostic factors for OS, and calculated hazard ratios (HRs). All data were analyzed using EZR software (Saitama Medical Center, Jichi Medical University, Japan). *P* < 0.05 was considered statistically significant.

## 3 RESULTS

### 3.1 Patient background

Table 1 shows the patients' characteristics. In total, 294 patients met the eligibility criteria, and they were divided into laparoscopy (*n* = 136 cases; 46.3%) and open (*n* = 158; 53.7%) groups. The median follow‐up period was 39 (interquartile range: 15–60) mo. Following PSM, there were 97 patients each in the laparoscopy and open groups. Before matching, the proportions of patients with total gastrectomy (*P <* 0.001) and patients with a preoperative CRP level of ≥0.5 were lower (*P =* 0.017) in the laparoscopy group. After matching, there were no significant differences in the patients' background characteristics.

**TABLE 1:** Patient characteristics before and after propensity score matching

	All patients				After matching		
	Laparoscopy group (*N* = 136)	Open group (*N* = 158)	*P* value		Laparoscopy group (*N* = 97)	Open group (*N* = 97)	*P* value
Sex							
Male	87 (64.0%)	114 (72.2%)	0.166		71 (73.2%)	65 (67.0%)	0.433
Female	49 (36.0%)	44 (27.8%)			26 (26.8%)	32 (33.0%)	
Age, mean ± SD	68.58 ± 11.68	67.65 ± 11.13	0.486		67.22 ± 11.28	67.33 ± 11.77	0.945
Body Mass Index, mean ± SD	22.80 ± 3.51	22.49 ± 3.55	0.452		22.95 ± 3.36	22.17 ± 3.46	0.113
Surgical procedure							
Distal gastrectomy	84 (61.8%)	67 (42.4%)	<0.001		53 (54.6%)	54 (55.7%)	
Proximal gastrectomy	9 (6.6%)	2 (1.3%)			4 (4.1%)	2 (2.1%)	0.802
Total gastrectomy	43 (31.6%)	89 (56.3%)			40 (41.2%)	41 (42.3%)	
Clinical stage							
II	35 (25.7%)	36 (22.8%)	0.586		21 (21.6%)	24 (24.7%)	0.734
III	101 (74.3%)	122 (77.2%)			76 (78.4%)	73 (75.3%)	
Comorbidity							
CKD	30 (22.1%)	23 (14.6%)	0.128		14 (14.4%)	16 (16.5%)	0.843
COPD	35 (25.7%)	34 (21.5%)	0.411		22 (22.7%)	19 (19.6%)	0.725
Diabetes	16 (11.8%)	25 (15.8%)	0.399		13 (13.4%)	14 (14.4%)	1.000
CHF	7 (5.1%)	4 (2.5%)	0.356		5 (5.2%)	2 (2.1%)	0.444
Preoperative CRP ≥0.5 (mg/dL)	18 (13.2%)	39 (24.7%)	0.017		16 (16.5%)	20 (20.6%)	0.580
SMI (cm^2^/m^2^), median (IQR)	39.13 (34.27–46.65)	38.81 (33.50–43.87)	0.433		39.59 (35.37–47.01)	38.69 (33.82–43.19)	0.136
Low‐SMI	65 (50.8%)	84 (54.9%)	0.549		44 (47.8%)	53 (55.8%)	0.307
VAI (cm^2^), median (IQR)	29.34 (19.24–48.72)	33.97 (13.62–52.16)	0.551		31.59 (20.64–47.12)	33.48 (13.05–51.55)	0.837
Low‐VAI	70 (54.7%)	79 (51.6%)	0.633		48 (52.2%)	50 (52.6%)	1.000

Abbreviations: CHF, chronic heart failure; CKD, chronic kidney disease; COPD, chronic obstructive pulmonary disease; CRP, C‐reactive protein; IQR, interquartile range; SD, standard deviation; SMI, skeletal muscle mass index; VAI, visceral adipose tissue index.

### 3.2 Postoperative outcomes after matching

Table 2 shows the postoperative outcomes after matching. The operating time was longer (*P <* 0.001), while intraoperative blood loss was lesser in the laparoscopy group (*P <* 0.001) than in the open group. There were no between‐group differences in the rates of total postoperative complications (*P =* 1.000) and severe postoperative complications (*P =* 0.164), pathological stage (*P =* 0.76), T factor (*P =* 0.493), lymph node metastasis (*P =* 0.734), and histological type (*P =* 1.000). Adjuvant chemotherapy was performed in 71.1% patients in both groups (*P =* 1.000), and the treatment completion rate was significantly higher in the laparoscopy group than in the open group (91.3% vs 78.3%; *P =* 0.032). The recurrence rate and mortality rate after surgery were significantly higher in the open group (*P =* 0.002 and *P <* 0.001, respectively). There were no significant differences in the BWL rate for 1 mo (*P =* 0.357), 6 mo (*P =* 0.629), and 1 y (*P =* 0.281).

**TABLE 2:** Postoperative outcomes after matching

	Laparoscopy group (*N* = 97)	Open group (*N* = 97)	*P* value
Operating time (min), median (IQR)	285.0 (235.0–340.0)	235.0 (200.0–280.0)	<0.001
Intraoperative blood loss (g), median (IQR)	20.0 (9.0–42.0)	165.0 (60.0–300.0)	<0.001
Postoperative complication			
Clavien‐Dindo classification ≥2	20 (20.6%)	20 (20.6%)	1.000
Clavien‐Dindo classification ≥3	7 (7.2%)	14 (14.4%)	0.164
Pathological stage			
II	37 (38.1%)	40 (41.2%)	0.769
III	60 (61.9%)	57 (58.8%)	
T factor			
T3	64 (66.0%)	57 (58.8%)	
T4a	31 (32.0%)	36 (37.1%)	0.493
T4b	2 (2.1%)	4 (4.1%)	
Lymph nodes metastasis			
Absent	21 (21.6%)	24 (24.7%)	0.734
N1	23 (23.7%)	22 (22.7%)	1.000
N2	25 (25.8%)	20 (20.6%)	0.497
N3	28 (28.9%)	31 (32.0%)	0.755
Histological type			
Differentiated	36 (37.1%)	37 (38.1%)	1.000
Undifferentiated	61 (62.9%)	60 (61.9%)	
All cases of adjuvant chemotherapy	69 (71.1%)	69 (71.1%)	1.000
Completion	63 (91.3%)	54 (78.3%)	0.032
Discontinuation	6 (8.7%)	15 (21.7%)	0.032
Recurrence after surgery	29 (29.9%)	50 (52.6%)	0.002
Lymph node metastasis	10 (10.3%)	18 (18.6%)	0.152
Liver metastasis	6 (6.2%)	10 (10.3%)	0.435
Peritoneal metastasis	18 (18.6%)	31 (32.0%)	0.047
Lung metastasis	0 (0%)	4 (4.1%)	0.121
All deaths	20 (20.6%)	46 (47.4%)	<0.001
Cancer‐specific death	13 (13.4%)	34 (35.1%)	0.001
Other‐cause death	7 (7.2%)	12 (12.4%)	0.334
Postoperative body weight loss (%)			
For 1 mo, median (IQR)	7.17 (4.93–10.55)	8.08 (5.81–11.65)	0.357
For 6 mo, median (IQR)	11.82 (6.78–16.80)	12.14 (6.28–16.51)	0.629
For 1 y, median (IQR)	12.06 (8.13–17.54)	10.82 (5.94–16.97)	0.281

Abbreviation: IQR, interquartile range.

### 3.3 Long‐term oncological outcomes after matching

**FIGURE 1:**
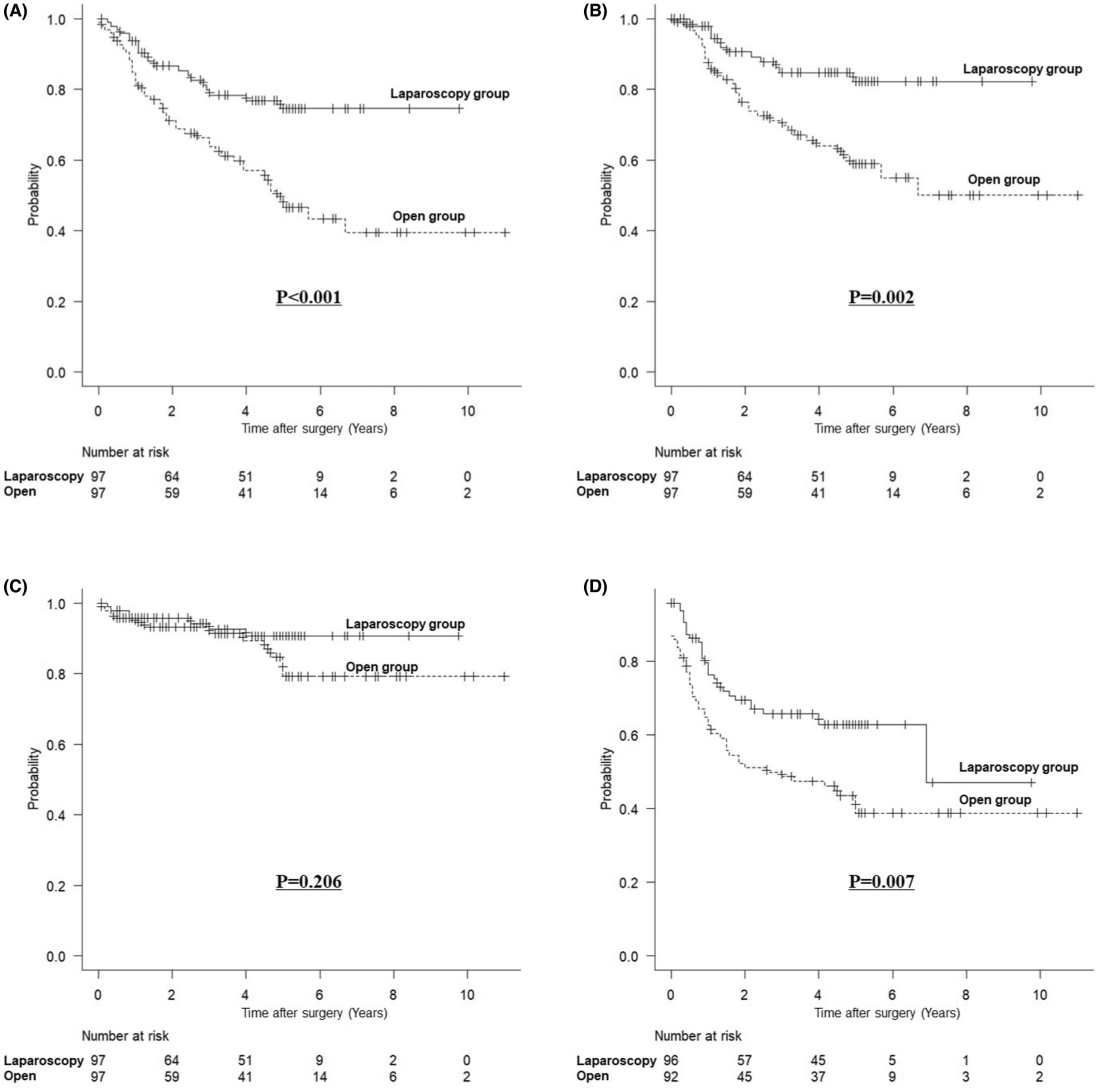
Kaplan–Meier survival curves according to surgical approach after matching. (A) For overall survival (*P <* 0.001). (B) For cancer‐specific survival (*P =* 0.002). (C) For other‐cause survival (*P =* 0.206). (D) For relapse‐free survival (*P =* 0.007)

**FIGURE 2:**
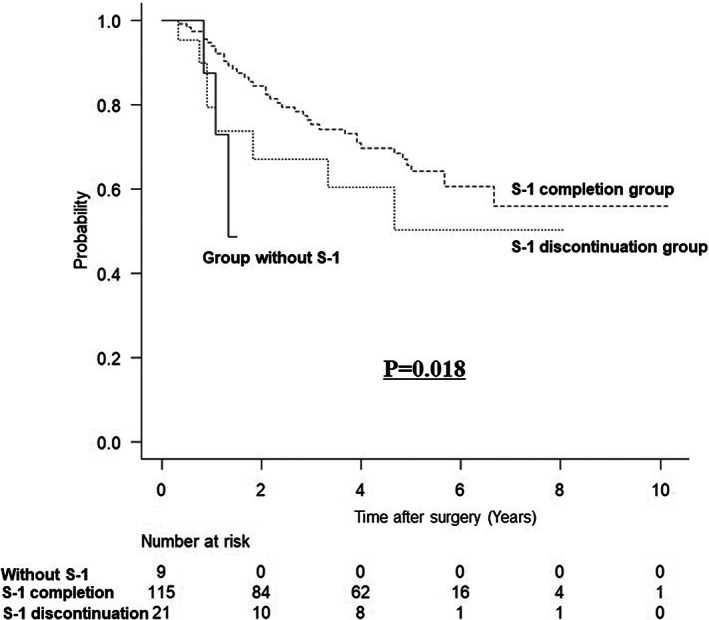
The overall survival based on the adjuvant chemotherapy administered after matching

Figure 1 shows the long‐term prognosis after matching. After matching, OS (HR: 2.449, 95% confidence interval [CI]: 1.448–4.142; *P <* 0.001; Figure 1A) and CSS (HR: 2.786, 95% CI: 1.469–5.282; *P =* 0.002; Figure 1B) were significantly worse in the open group than in the laparoscopy group. However, there was no between‐group difference in OCS after matching (HR: 1.825, 95% CI: 0.718–4.637; *P =* 0.206; Figure 1C). RFS was significantly worse in the open group than in the laparoscopy group (HR: 1.809, 95% CI: 1.175–2.784; *P =* 0.007; Figure 1D). Figure 2 shows the OS based on the adjuvant chemotherapy administered after matching. OS was better in the order of S‐1 completion group, S‐1 discontinuation group, and group without S‐1 (*P =* 0.018).

### 3.4 Prognostic factors for OS

The results of multivariate analysis with a forward stepwise procedure of the prognostic factors for OS in all patients are shown in Table 3. Multivariate analysis showed that age ≥70 y (HR: 2.279, 95% CI: 1.423–3.651, *P =* 0.001), open surgery (HR: 2.160, 95% CI: 1.365–3.419, *P =* 0.001), serosal invasion (HR: 1.853, 95% CI: 1.209–2.840; *P =* 0.005), lymph node metastasis (HR: 3.122, 95% CI: 1.667–5.846, *P <* 0.001), preoperative CRP ≥0.5 mg/dL (HR: 1.665, 95% CI: 1.009–2.749, *P =* 0.046), and low‐VAI (HR: 1.847, 95% CI: 1.186–2.874, *P =* 0.007) were independent prognostic factors.

**TABLE 3:** Results of analyses of prognostic factors for overall survival in all patients

Variables	Multivariate analysis		
	HR	95%CI	*P* value
Age (y)			
<70	1		
≥70	2.279	1.423–3.651	0.001
Surgical approach			
Laparoscopic surgery	1		
Open surgery	2.160	1.365–3.419	0.001
Serosal invasion			
Absent	1		
Present	1.853	1.209–2.840	0.005
Lymph node metastasis			
Absent	1		
Present	3.122	1.667–5.846	<0.001
Adjuvant chemotherapy			
Absent	1		
Present	0.668	0.399–1.119	0.126
Diabetes			
Absent	1		
Present	1.518	0.873–2.640	0.139
Preoperative CRP			
<0.5 mg/dL	1		
≥0.5 mg/dL	1.665	1.009–2.749	0.046
Postoperative complication			
Absent	1		
Clavien–Dindo ≧3	1.706	0.951–3.062	0.073
VAI (cm^2^/m^2^)			
High‐VAI	1		
Low‐VAI	1.847	1.186–2.874	0.007

Abbreviations: CI, confidence interval; CRP, C‐reactive protein; HR, hazard ratio; VAI, visceral adipose tissue index.

## 4 DISCUSSION

In this study, laparoscopic surgery and open surgery were compared after adjustment with PSM in patients who underwent radical gastrectomy for primary T3 or more advanced gastric cancer. After matching, OS, CSS, and RFS were significantly better in the laparoscopy group. The adjuvant chemotherapy completion rate was significantly higher in the laparoscopy group than in the open group, while the recurrence rate and mortality rate after surgery were significantly higher in the open group. Multivariate analyses also showed that open surgery was an independent poor prognostic factor for OS in patients with primary T3 or more advanced gastric cancer.

A primary reason for poor OS, CSS, and RFS in the open group was poor compliance with postoperative adjuvant chemotherapy. Bao et al reported that laparoscopic surgery facilitated better compliance with postoperative adjuvant chemotherapy than did open surgery; moreover, it improved the long‐term prognosis.^12^ The authors attribute this to the fact that laparoscopic surgery is less invasive and postoperative recovery is faster. The ACTS‐GC trial showed that the time from relapse to death did not differ between surgery with adjuvant S‐1 chemotherapy and surgery alone,^13^ and that differences in compliance may not affect the time from relapse to death. Therefore, good compliance with laparoscopic surgery may contribute to differences in RFS, which may directly correlate with OS and CSS. We also reported that laparoscopic surgery may prolong RFS compared to that after open surgery in patients with gastric cancer showing reduced muscle loss.^14^ A meta‐analysis has shown that the side effects of chemotherapy are more severe in patients with reduced muscle loss.^15^ In this study, more than half the patients had low SMI, as defined by the cutoff values for Asians; this may have resulted in the stronger prognostic impact of laparoscopic surgery. These results suggest that the prevalence of sarcopenia increases with the stage of cancer, with greater benefits of laparoscopic surgery. In the CLASS‐01 trial, the percentage of postoperative adjuvant chemotherapy was lower at about 40%.^1^, ^3^ In this study, 70% of patients received postoperative adjuvant chemotherapy, which may have increased the prognostic difference because of the high demand for postoperative adjuvant chemotherapy.

The second reason for poor RFS in the open group was the systemic immune response. Buunen et al^16^ reviewed the systemic immune response to a CO_2_ pneumoperitoneum, with particular reference to inflammatory cytokine levels and T‐cell function, and noted that laparoscopic surgery had a smaller effect on systemic immunity than open surgery. This difference may be related to cancer development, and the more advanced the cancer, the greater the difference may be.

With regard to the recurrence type, peritoneal metastasis was significantly more common in the open group in the present study, while other types of metastases showed no difference. A systematic review of high‐quality non‐RCT studies of advanced gastric cancer found a trend toward fewer recurrences with laparoscopic surgery than with open surgery. In particular, there were fewer local recurrences and no difference in peritoneal recurrences.^17^ The ACTS‐GC trial showed that S‐1 adjuvant chemotherapy was effective in reducing peritoneal recurrence,^18^ and the better compliance with adjuvant chemotherapy in the laparoscopy group in the present study may be a reason for the difference in peritoneal recurrence.

This study had several limitations. First, it was a single‐center, retrospective cohort study. Second, at the time of treatment selection there was a possibility of selection bias in terms of the surgical procedure. Before matching, the rate of total gastrectomy was significantly higher in the open group. A previous study using PSM showed that OS was better in the laparoscopy group before matching; however, the difference disappeared after matching.^6^ This may be attributed to selection bias introduced by the surgeon, who unintentionally assigned patients in better condition to laparoscopic surgery. To avoid selection bias, the present study was limited to patients with pathological T3 or deeper cancer, and the long‐term prognosis was compared after adjustment for background characteristics using PSM. The results showed no differences in the surgical procedure and pathological features. Third, for quality control we only involved a single surgeon who was a professional in gastrectomy in the present study. Therefore, there was no difference in the quality of surgery. In actual clinical settings, the quality of surgery would differ among surgeons, and the results could be different. Fourth, the pathogenesis‐based reason for the difference in long‐term prognosis between open and laparoscopic surgery is unclear. Further research is needed, including basic data on the effects of the laparoscopic surgical environment with CO_2_ pneumoperitoneum on cancer development and recurrence.

In conclusion, overall survival may be better with laparoscopic gastrectomy than with open surgery for patients with primary T3 or more advanced gastric cancer. To our knowledge, this is the first study to show the impact of minimally invasive surgery on the long‐term prognosis of patients with T3 or more advanced gastric cancer. The results are expected to form the basis for future studies.

## AUTHOR CONTRIBUTIONS

R. Matsui and N. Inaki equally contributed to the conception and design of the research; R. Matsui and T. Tsuji contributed to the acquisition and analysis of the data; R. Matsui and N. Inaki contributed to the interpretation of the data; and R. Matsui and N. Inaki drafted the article. All authors critically revised the article, and agreed to be fully accountable for ensuring the integrity and accuracy of the work, and read and approved the final article. The study protocol was approved by the Institutional Ethical Review Committee of Ishikawa Prefectural Central Hospital (authorization number: 1589). The opt‐out recruitment method was applied to provide all patients an opportunity to refuse participation.

## FUNDING INFORMATION

This research did not receive any specific grant from funding agencies in the public, commercial, or not‐for‐profit sectors.

## CONFLICT OF INTEREST

The authors declare no conflicts of interest.

## HUMAN RIGHTS STATEMENT AND INFORMED CONSENT

All experimental protocols described in this study were approved by the Institutional Ethical Review Committee of the Ishikawa Prefectural Central Hospital (authorization number: 1589). All procedures followed were in accordance with the ethical guidelines of Japan's Ministry of Health, Labor, and Welfare for medical and health research involving human subjects and with the Helsinki Declaration of 1964 and later versions. The opt‐out recruitment method was applied to provide all patients an opportunity to decline to participate.

## ETHICAl APPROVAL

The study protocol was approved by the Institutional Ethical Review Committee of Ishikawa Prefectural Central Hospital (authorization number: 1589). The opt‐out recruitment method was applied to provide all patients an opportunity to refuse participation.

## Data Availability

The datasets generated during and/or analyzed during the current study are available from the corresponding author on reasonable request.
